# Credulity and Credibility

**Published:** 1916-01-22

**Authors:** 


### CREDULITY AND CREDIBILITY.

### By a Medical Officer from France.

Two of the things that most strike the ordinary man returned from the Front to England are the recruiting posters and the amazing credulity of everyone. The most ridiculous rumours circulate freely, are everywhere believed, and pass from mouth to mouth with astonishing celerity. The Dardanelles were conquered three times a week and Lille was taken once a fortnight by London gossip; and the discovery that each such rumour is a lie never seems to depress the story-mongers nor to imbue them with any sense of scepticism. Many of the stories repeated with childlike faith by all classes of the community are themselves so self-contradictory, so inconsistent on the very faces of them, that one wonders how any educated man or woman can be got to repeat them or to believe them for a moment. In France, goodness knows, there are plenty of lying rumours to be heard in the trenches; but they are treated at their true value as idle gossip.

### Mr. Atkins as a Raconteur.

Given this extraordinary, unreasoning, indiscriminating credulity of the average Englishman about the war—and that of the Englishwoman is even greater—it is not surprising that that prince of *raconteurs* and very splendid fellow Mr. T. Atkins has achieved some wonderful flights of fancy. Possibly one reason for the cautiousness of the average officer in France in accepting for revealed truth every yarn that goes the round of the mess is the insight which he gets, while censoring letters, into the romancing tendencies of the private soldier. It is not only that a percentage of the men in the ranks retail to their friends every report that they chance to hear, and even embroider them for home consumption: just as often they narrate incidents that are purely imaginary, apparently for the purpose of giving their womenfolk thrills of horror and anxiety, or of extorting the admiration and envy of their male friends. Thus on one occasion, to give an instance which any officer could cap without difficulty, a soldier arrived fresh out from England in a reinforcing draft, and was duly posted to a unit which happened to be about four miles behind the trench line. He was allotted certain clerical duties which kept him fairly well employed indoors, and certainly gave him no chance of exploration nearer the fire trenches. Four days after his arrival, during which time no shell had fallen within two miles of him, he wrote home as follows:—

"We are now in the trenches, seeing the bullets doing their ghastly work and hearing the constant scream of the shells overhead. But I am a soldier and no coward; and I do my duty just the same."

It would not be fair to suggest that all the wonderful stories told in letters from the trenches have as little foundation as in this case; for even the most extraordinary have generally some basis in fact, though the men who do the most wonderful feats of bravery and heroism are apt to be the last to write about them and the most taciturn when they do refer to their own adventures. An authentic instance of an almost pardonable exaggeration will show how an honest mistake combined with a vivid imagination may result in a story which is as misleading as a deliberate tissue of lies. In the autumn of 1914 the *Daily Mail* printed a letter written by a private soldier concerning his experiences at the battle of the Aisne in mid-September. From the name and number of the battalion to which he belonged it was quite certain that he was serving in a certain division which lost very heavily. At the time referred to in the narrative there were (across the river) but two dressing-stations for the whole of this division: one was a small one in a barn, the other a very large one in a big church and certain adjacent buildings. The soldier wrote a graphic and manly account of the fighting, of his sensations when wounded, and of his adventures while journeying from the trenches to the dressing-station. He gave a good and clear account of the scene in a church, and of various topographical points connected with it; it was evident that he had been admitted as a patient to the particular church, which was the only one in use for that division. He then described how he lay on a mattress on the altar steps: this, again, is quite credible, for the church was so crowded with wounded that of necessity several mattresses had to be laid there. Finally, he stated that a German shell struck the other end of the church, blew a hole through the wall, and killed fifty of the wounded as they lay. This was where the inaccuracy crept in. A shell did enter the west end of the church, made a big hole in the wall, and filled the building with smoke and dust. But, as luck would have it, that end of the church had just been cleared of the wounded, and not a single man was hurt by the explosion. Yet any critic, judging on internal evidence alone, would have been justified in declaring this letter to be an honest and credible account of facts within the soldier's actual knowledge and experience: that one detail about the fifty lost lives was the only inaccuracy in a very vivid and very straightforward story.

### The Story of the Angels.

The lesson is that the undue credulity of those at home and the overrated credibility of the soldier abroad have resulted in some strange stories. Take the celebrated angels, or bowmen, or whatever they are asserted to be, of Mons. This story, invented as a piece of pure fiction by Mr. Machen, is now being repeated in many versions by various private soldiers, who may or may not have convinced themselves by repetition that they did actually see certain supernatural beings. Angels in themselves are credible enough, and for many people at home the fact that they believe in angels seems sufficient reason that they should therefore believe in these particular angels. There are, however, certain points which the unthinking and the overcredulous seem to overlook. First, they accept as literally and absolutely true certain very vague and mutually inconsistent statements made months after the battle by private soldiers: they make no attempt to estimate the credibility of the witnesses. Next, they overlook the fact that no one ever heard of these interesting occurrences before the publication of Mr. Machen's story; nor, indeed, until long after. Yet surely a *raconteur* of Mr. Atkins' abilities would never have neglected to tell his friends about them while they were fresh in his mind? Next, they pay a poor compliment both to the British Army and to the hosts of heaven if the two combined could not stay even for a day the victorious German advance. Contemptible indeed must Sir John French's gallant command have seemed to the Kaiser if such were the case. Nor were the angels defeated once only, according to the "evidence" which the credulous accept without stopping to examine it.

